# Cryopreservation of the Mediterranean fruit fly (Diptera: Tephritidae) VIENNA 8 genetic sexing strain: No effect on large scale production of high quality sterile males for SIT applications

**DOI:** 10.1371/journal.pone.0211259

**Published:** 2019-01-25

**Authors:** Ihsan ul Haq, Adly Abd-Alla, Ulysses Sto Tomas, Jose Salvador Meza, Kostas Bourtzis, Carlos Cáceres

**Affiliations:** 1 Insect Pest Control Laboratory, Joint FAO/IAEA Division of Nuclear Techniques in Food and Agriculture, Seibersdorf, Austria; 2 National Agricultural Research Centre, Park Road Islamabad, Pakistan; 3 Programa Moscafrut, SAGARPA-IICA, Metapa de Domínguez, Chiapas, México; College of Agricultural Sciences, UNITED STATES

## Abstract

The sterile insect technique (SIT) integrated in area-wide integrated pest management (AW-IPM) programmes is being used for the successful management of the Mediterranean fruit fly *Ceratitis capitata* (Wiedemann) (Diptera: Tephritidae) which is a horticultural pest of economic importance in tropical and subtropical countries. All programmes with an SIT component are using the VIENNA genetic sexing strains (GSS), mainly the VIENNA 8 GSS, which have been developed by applying classical genetic approaches. The VIENNA 8 GSS carries two selectable markers, the white pupae and the temperature sensitive lethal genes, which allows the production and release of only males thus increasing the biological efficiency and cost effectiveness of SIT applications. However, mass rearing may affect quality traits of the GSS, in which case replenishment of the colony with wild flies is recommended, a process which is tedious and time consuming. We previously reported the development of a cryopreservation protocol for the VIENNA 8^D53+^ strain. In the present study, we report on the evaluation of the cryopreserved strain VIENNA 8^D53+^/Cryo-228L, reared under semi mass rearing conditions, for production parameters, quality control indices and mating competitiveness of males, in a comparative way with the non-cryopreserved VIENNA 8^D53+^ strain, against wild type males. The VIENNA 8^D53+^ and VIENNA 8^D53+^/Cryo-228L strains were similar for production parameters viz. egg production, pupal production, pupal recovery, and quality control indices like fly emergence, sex ratio and flight ability. Males from both strains were equally competitive with males of the wild type strain in achieving mating with wild type females under field cage conditions. Results are discussed in the context of cryopreservation as a potential backup strategy for refreshing the mass rearing colony with biological material from a cryopreserved stock.

## Introduction

The Mediterranean fruit fly *Ceratitis capitata* (Wiedemann) (Diptera: Tephritidae) is one of the most destructive pests in the world, causing extensive direct damages to horticultural produces and indirect losses by impeding the international trade [1,2]. It is extremely polyphagous, and attacks more than 350 species of fruits and vegetables [3,4]. Application of synthetic insecticides in addition to causing damages to the environment remained insufficient to suppress this pest population to a level that can meet the quarantine requirements. Therefore, management of this pest necessitates the application of strategies which are environmentally friendly and effective to suppress the population to a desired level [5–7]. The sterile insect technique (SIT) is an eco-benign technique which relies on the mass-rearing of insects of the target population, sterilizing them by ionizing radiation and releasing them in the target areas where released sterile mass-reared males mate with virgin wild females and transfer their sterile sperm which results in no off-springs [8]. Successive and sustained releases of sterile males can gradually reduce the density of the target population to a very low, economically acceptable level and, in some cases, eradication can also be achieved [8]. The SIT has been proven very effective for the suppression, containment, prevention or eradication of populations of the Mediterranean fruit fly [9]. Two main factors have made the SIT for the Mediterranean fruit fly more cost-effective: first, the technological developments that enabled the production of flies on a large scale i.e. more than 4000 million sterile pupae per week are produced in different facilities world-wide [10] and second, the development of genetic sexing strains (GSS) [9] which allowed the separation of sexes early in development. The Mediterranean fruit fly ‘first generation’ GSS was based upon a pupal color mutation that allowed the separation of male brown pupae (wild type) from female white pupae (due to a mutation in the *wp* gene) [11]. The ‘second generation’ GSS carried an additional mutation in the temperature sensitive lethal (*tsl*) gene [12,13]. Female embryos homozygous for the *tsl* mutation are sensitive to temperature and die upon exposure to 34 ºC for 24 h. Therefore, the GSS carrying the mutations in the *wp* and *tsl* genes allows the production and release of males only. This improves the cost effectiveness and the biological efficiency of the SIT application due to the following reasons: (i) male-only releases eliminated the assortative mating between sterile males and sterile females, making the SIT component much more effective [14], (ii) dispersal of the sterile males to find wild females for mating is higher which enabled them to transfer their sterile sperms to more wild females and (iii) male-only releases reduced the cost of releasing sterile males [15,16]. In view of these advantages, all operational programs applying the SIT for management of this pest are rearing the GSS of this species [17,18]. Despite the fact that GSS are performing very well in small scale rearing environments, they may be vulnerable to break down, due to recombination phenomena, under large scale mass rearing conditions [19]. To overcome this potential problem, the colony management practice i.e. filter rearing system (FRS) was introduced. The FRS relies on maintaining a small standby colony of GSS under less crowded conditions, with no recombinants, which can regularly refresh the mainstream of production with new material. The key factor of this practice is that no flies from the mainstream colony are returned to the filter colony [20]. An additional step for preventing recombination-related problems was the integration of an inversion, known as D53, on some versions of the VIENNA 8 GSS [13,18].

The SIT incorporated as a component of AW-IPM programs has been remarkable for the successful population management of different insect pest species [21]. However, an important factor that undermines the quality and mating competitiveness of mass-reared males is the mass rearing environment itself. Mass rearing of flies continuously under crowded conditions has been shown to alter the mating behavior of mass reared, for instance, Mediterranean fruit fly and the melon fly males [22]. The strategies adopted to overcome the reduced mating competitiveness of mass reared males were either by increasing the over-flooding ratio of sterile to wild males or improving the quality of mass-reared males by refreshing the mass-reared colonies with wild flies. Refreshing the mass reared colony of a GSS is by far more difficult and time consuming (it may take over a year) than refreshing a mass reared colony of a bisexual strain. This is because of refreshing a GSS requires a series of genetic crosses and special attention for not losing the selectable markers, which are key genetic features of these strains, during the introgression of the new genetic background.

Due to the above mentioned limitations and challenges, cryopreservation has been considered as an alternative tool for refreshing the strains when needed [23,24]. The cryopreservation technology has been successfully demonstrated in a few economically important insect pest species such as the New World Screwworm *Cochliomyia hominivorax* [25], the Caribbean fruit fly, *Anastrepha suspensa* [26], the Mexican fruit fly, *Anastrepha ludens* [27], and the wild Mediterranean fruit fly [28]. Recently, we developed a modified cryopreservation protocol for the Mediterranean fruit fly VIENNA 8^D53+^ GSS, which is currently used in mass rearing and SIT applications, and we showed that, the cryopreservation does not affect the genetic structure and the productivity under laboratory (small scale) rearing conditions [24]. In the present study, we compared the productivity and key quality control parameters for SIT applications, including male mating competitiveness, of the cryopreserved strain VIENNA 8^D53+^/Cryo-228L, reared under semi mass rearing conditions, in a comparative way with the non-cryopreserved VIENNA 8^D53+^ strain.

## Materials and methods

### Strains

The VIENNA 8^D53+^ strain is a GSS that was developed at the FAO/IAEA Insect Pest Control Laboratory (IPCL), Seibersdorf, Austria and carries two selectable markers, white pupae (*wp*) and temperature sensitive lethal (*tsl*) genes, and the D53 inversion [13]. The VIENNA 8^D53+^/Cryo-228L strain was developed at the FAO/IAEA Insect Pest Control Laboratory (IPCL), Seibersdorf, Austria by cryopreservation of embryos of the VIENNA 8^D53+^ strain as described previously [24]. Both strains at the time of evaluation were 22 generations under small scale laboratory rearing conditions. The wildish strain originated from Argentina was maintained at the IPCL and at the time of evaluation it was 5^th^ generation under laboratory rearing. This strain did not carry any morphological marker, or other mutation, and it was used as reference for the mating behavioral assessment of the VIENNA 8^D53+^ and VIENNA 8^D53+^/Cryo-228L strains.

### Rearing protocol

The two Mediterranean fruit fly GSS (VIENNA 8^D53+^ and VIENNA 8^D53+^/Cryo-228L) were reared under a semi mass-rearing environment at the IPCL. Around 300,000 pupae at a 1:1 male:female ratio were placed in standard mass-rearing cages. These cages are of aluminum frame (201 (long) × 100 (high) × 20.5 cm (wide)) with both sides covered with muslin cloth net for female oviposition. These cages (bottom 45 cm up from the floor level) were hung from the roof side-by-side, 200 cm from the wall and 100 cm apart (each cage was suspended half-way between lights) in a room at 24 ± 1 ºC and 60 ± 5% RH with photoperiod 12:12 D/L. Adults were provided with a diet containing hydrolysate yeast as a source of protein and sugar in a ratio of 1:3 by weight respectively and water *ad libitum*. Females laid eggs by inserting their ovipositor through the muslin cloth net. Eggs dropped down in water in the iron toughs which were placed at the bottom of cages. Eggs were sieved out daily from the water and incubated in aerated water for 48 h at 24 ºC [29,30]. After incubation, the eggs were transferred to a standard larval diet [31] that contained wheat bran as the bulking agent. A volume of 3.5 mL of eggs (homogeneously mixed in water) was transferred to 5 kg larval diet in standard plastic larval Hawaiian rearing trays (77 × 40 × 3.8 cm (length–width—height) and corners height was 7 cm. The difference in height of tray sides and corners allows stacking them on top of each other leaving a gap of 3.2 cm between the surface of the diet in one tray and the bottom of the next tray, providing essential ventilation for the developing larvae and adequate space for the mature larvae to “pop out” of the diet for pupation [32]. Larvae pupated in saw dust in metal trays placed at the bottom of larval trays were collected. The same rearing protocol was used for both GSS that were reared in parallel and in the same insectary.

The wildish flies were reared in 30 × 30 × 45 cm plexiglass cages that had openings for manipulations that were covered with muslin cloth. The adult feeding protocol was the same as for the GSS and eggs were collected in plastic channels containing water placed alongside the cages. The females inserted their ovipositors through the screen net for egg laying and these eggs were dropping down in the water and were sieved out. The larval diet was also the same as used to feed the GSS larvae, but the larval plastic trays were smaller (30 × 40 cm).

### Egg and pupa production

Eggs produced by each strain were collected for 2 weeks and their volume was assessed. Eggs were transferred to the larval diet for pupae production for five times and in each transfer eggs were transferred to 3 larval trays. Pupae production from both strains was measured in volume. Egg to pupal recovery was calculated by dividing the number of pupae (1 L contains 56000 pupae) by the number of eggs (1mL contains 26000 eggs).

### Quality control indices

The quality control indices i.e. adult emergence, sex ratio, and flight ability were assessed by following the standard procedures [33]. A sample (5 mL) of pupae was taken from each collection and the pupae were sexed by sorting out the brown (male) from white (female) pupae. Sex ratio was determined by counting the number of males and dividing this number by the number of females. The incidence of recombinants from the sample of 5 mL pupae was monitored by observing that emerged flies are true to their pupal color. Adult emergence (%) was observed from the sample of 5 mL pupae and was calculated by dividing the number of adults emerged out of the number of pupae and multiplied by one hundred. Adult emergence for males and females was calculated separately. For flight ability, a sample of 5 mL pupae was taken and flight ability of adults from brown and white pupae was assessed separately. The pupae were placed within the ring of paper (1cm wide and 4 cm in diameter), which was centred in the bottom of the Petri dish two days before emergence. A 3-mm thick walled PVC tube (8.9 cm diameter × 10 cm height) was placed on the bottom of petri-dish. Before use, the inside of the tube was lightly coated with unscented talcum powder to avoid flies crawling out of the cylinder. To provide a resting place for newly emerged flies, talcum powder up to 1 cm of height was removed from the bottom of the tube. After all flies had emerged, the number of emerged flies, non-emerged pupae, and flies that had flown out of the tube were counted. The flight ability test was carried out in a glass chamber placed in a separate room with temprature 25 ± 1°C and 65 ± 15% RH. Five replications for each of the QC indices were evaluated.

### Field cages

The field cages used for the mating competitiveness trials were standard screened, circular field tents (2.2 m high × 2 m diameter) [33,34], containing a potted citrus tree of ~2 m height. Five such field cages were placed inside a large insect greenhouse (24 × 10 × 4 m) that allowed us to carry out 5 replicates of the test simultaneously. A temperature of 25 ± 1°C and 50 ± 5% RH was maintained throughout the experiment. Field cage tests took place under a semi-natural illumination and photoperiod provided by a translucent roof of the insect greenhouse.

### Mating competitiveness tests in field cages

Mating competitiveness tests were performed in walk-in field cages adopting standard protocols [33]. A sample of ~300 pupae was taken from the stock produced under semi mass-rearing conditions and transferred to cylindrical plexiglass screened cages (30 cm height and 64 cm diameter) and kept in the insectary where the wildish flies were maintained. Wildish flies were sexed 1–2 days after emergence and males and females maintained in separate cages. All flies of all strains that were used for the mating competitiveness studies were kept at insectary conditions of 23 ºC and 65 ± 5% RH and were provided with a protein diet and water *ad libitum*. Males from each strain were marked with different water colors (Faber-Castell; http://www.fabercastell.com) using a camel brush and the mark was applied on the dorsal side of the thorax of males confined by a screen net. Allocation of marking was rotated among treatments to avoid any effect of color preference by females. Male marking was done 1–2 days before the mating trials. Twenty males of each strain competed for twenty wild females at a male:female ratio of 3:1. All males were released in field cages early in the morning at 8:30 AM and 20 minutes later the females were released, and allowed to complete copulation under observation (every 15 min). The recorded parameters included number of matings and male type (identified by the respective color).

### Data analysis

The data on quality control indices were first analyzed for parametric assumptions and it showed normal distribution. The differences in production and quality control indices between two strains were analyzed by unpaired *t*-test. The data on mating success didn’t fulfill the parametric assumptions (Shapiro-Wilk normality test, W = 0.82, *P* = 0.008) and the difference among treatments was analyzed by Kruskal-Wallis test and complementary Dunn’s multiple comparison test [35]. The significant value used in data analysis was 95% (α = 0.05).

## Results

### Egg productivity under semi mass-rearing conditions

Both strains, VIENNA 8^D53+^ and VIENNA 8^D53+^/Cryo-228L, did not present any statistically significant differences as concerns the volume of eggs produced (*t* = 0.82, *P* = 0.42), egg hatch (*t* = 0.45, *P* = 0.65), pupal production (t = 0.61, *P* = 0.55) and egg to pupal recovery (t = 0.61, *P* = 0.55) as shown in Table 1.

**Table 1:** Egg production, egg hatch (%), pupal production, and pupal recovery (%) of the mediterranean fruit fly VIENNA 8^D53+^ and VIENNA 8^D53+^/Cryo-228L strains reared under semi mass rearing conditions. The comparison of each parameter is given in each column and non-significantly different (*t*-test, *P* > 0.05) parameters are represented by the letters ns.

Strains	Egg production (mL)	Egg hatch (%)	Pupal production (mL)	Pupal recovery (%)
**VIENNA 8**^**D53+**^	103 ns	66.13 ± 2.85 ns	8560 ns	35.12 ± 3.01 ns
**VIENNA 8**^**D53+**^**/Cryo-228L**	136 ns	64.67 ± 1.43 ns	9320 ns	38.24 ± 4.06 ns

### Quality control indices

There was no statistically significant difference between the VIENNA 8^D53+^ and VIENNA 8^D53+^/Cryo-228L strains in respect to male fly emergence (*t* = 1.33, *P* = 0.21), female fly emergence (*t* = 0.51, *P* = 0.62), male flight ability (*t* = 0.75, *P* = 0.47), female flight ability (*t* = 0.80, *P* = 0.44) and sex ratio (*t* = 1.15, *P* = 0.28) (Table 2).

**Table 2:** Quality control indices (Adult emergence, flight ability, and sex ratio) of the mediterranean fruit fly VIENNA 8^D53+^ and VIENNA 8^D53+^/Cryo-228L strains reared under semi mass rearing conditions. The comparison of each parameter is given in each column and non-significantly different (*t*-test, *P* > 0.05) parameters are represented by the letter ns.

	Adult emergence % (Mean ± SE)		Sex ratio	Flight ability % (Mean ±SE)	
Strains	Male	Female	Male : female	Male	Female
**VIENNA 8**^**D53+**^	81.51 ± 1.59 ns	65.07 ± 7.25 ns	1.25 : 1± 0.22 ns	96.08 ± 1.121 ns	80.38 ± 5.69 ns
**VIENNA 8**^**D53+**^**/Cryo-228L**	85.32 ± 2.36 ns	59.14 ± 8.98 ns	1.79 : 1 ± 0.41 ns	94.40 ± 1.91 ns	85.77 ± 3.47 ns

### Mating competitiveness

**Fig 1:**
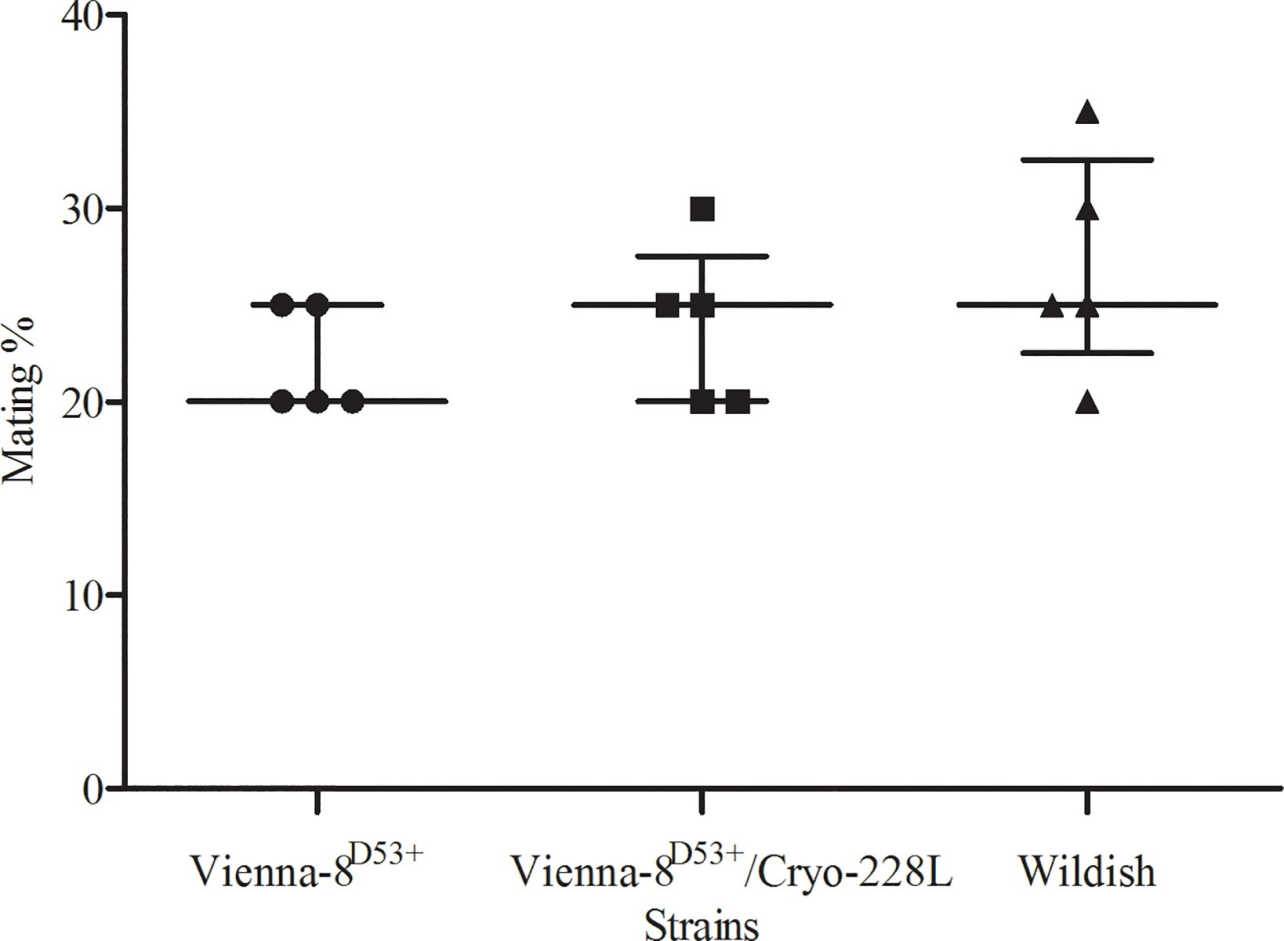
Mating success percentage (median ± interquartile range) by males of the Mediterranean fruit fly VIENNA 8^D53+^, VIENNA 8^D53+^/Cryo-228L and wildish strains competing for mating with wildish females (3:1) under field cage conditions. Medians are not significantly different from each other (Dunn’s Multiple Comparison Test, *P* > 0.05).

There was also no statistically significant difference in the mating success of males from the VIENNA 8^D53+^, VIENNA 8^D53+^/Cryo-228L and wildish strains (Fig 1) when competing for mating with wildish females (Kruskal-Wallis statistic = 2.65, *P* = 0.265).

## Discussion

The VIENNA 8^D53+^ and VIENNA 8^D53+^/Cryo-228L strains were similar in egg production, pupal production, pupal recovery as well as in quality control indices such as fly emergence, sex ratio, and flight ability. Males from both strains were equally competitive with males of the wildish strain for achieving mating with wildish females under field cage conditions. These data are in accordance with the findings of previous studies on other strains of *Ceratitis capitata* as well as on strains of *Anastrepha suspensa* and *Anastrepha ludens* [26], indicating that cryopreservation does not adversely affect the quality of the medfly GSS and they clearly suggested that cryopreservation can be used as a tool to preserve valuable strains such as the medfly VIENNA genetic sexing strains used for SIT applications worldwide.

The quality of the cryopreserved VIENNA 8^D53+^/Cryo-228L strain was previously tested under small scale rearing conditions with no evidence of negative effects [24]. However, the mass rearing conditions are generally more stressful than small scale ones and this necessitated further assessment on the productivity and the quality of the strain under a large scale setting. The flies in mass rearing are typically cultivated under crowded conditions which often results in genetic changes with subsequent changes in their behavior. Although it is difficult to assess the factors that drive these changes, however, bottleneck, inbreeding or genetic drift have previously been reported [36,37]. In addition, changes in the associated symbiotic community may occur due to the artificial rearing and conditions which may affect the mating behavior and other fitness traits of flies [38]. Therefore, it was imperative to assess the production parameters and the quality, including the male mating competitiveness, of flies reared under mass-rearing conditions.

For successful application of SIT, the sustained production of competitive sterile males is required. However, mating competitiveness of mass reared males may be compromised due to the mass rearing environment and the poor male mating competitiveness can cause negative consequences to the effectiveness of SIT application. For instance, this was the case with the melon fly eradication program in Okinawa prefecture, Japan. The project was going on satisfactorily when monitoring and recapture of sterile males started showing that the efficiency of the eradication program was gradually declining, mainly due to the inferior mating competitiveness of mass reared males as compared with wild males. Further investigations showed that the inferior quality of mass reared males could not be detected in mating trials under field cage conditions but mass reared sterile males were clearly far less competitive than wild males in the field [39]. To rectify such potential problems, one approach could be to increase the over-flooding ratio of sterile males but this would increase the cost of the program. Alternatively, it would require the *de novo* production of competitive sterile males, and this was the strategy implemented in the melon fly eradication program through the routinely refreshment of the mass reared colony, which was a bisexual strain, with wild flies [39]. However, refreshing the colony of a GSS is a rather tedious and time consuming approach and in this scenario, cryopreservation may play an important role by preserving the remarkable biological material. Thus, reviving the GSS bearing the desired traits i.e. higher productivity and higher male mating competitiveness, can save a lot of time and costs to operational programmes.

Furthermore, cryopreservation may also be a strategy to conserve diverse genetic material used for strain improvement or to maintain large number of mutant lines or cryopreserving a strain for quick production when needed. For instance the screwworm fly has been eradicated form North and Central America, and North Africa and the facilities for rearing of screwworm, except one facility in Panama, have been shut down. If resurgence of fly happened, then cryopreserved flies can be reared, multiplied and can be utilized to suppress the introduction of fly.
